# Need for speed: Human fast-twitch mitochondria favor power over efficiency

**DOI:** 10.1016/j.molmet.2023.101854

**Published:** 2023-12-15

**Authors:** Sebastian Edman, Mikael Flockhart, Filip J. Larsen, William Apró

**Affiliations:** 1 Department of Women's and Children's Health, Karolinska Institute, Stockholm, Sweden; 2 The Åstrand Laboratory, Department of Physiology, Nutrition and Biomechanics, The Swedish School of Sport and Health Sciences, Stockholm, Sweden; 3 Department of Public Health and Clinical Medicine, Umeå University, Umeå, Sweden; 4 Department of Clinical Sciences, Intervention and Technology, Karolinska Institute, Stockholm, Sweden

**Keywords:** Muscle fiber types, Mitochondria, Respiration, Electron transport chain, Human, Succinate

## Abstract

**Objective:**

Human skeletal muscle consists of a mixture of slow- and fast-twitch fibers with distinct capacities for contraction mechanics, fermentation, and oxidative phosphorylation. While the divergence in mitochondrial volume favoring slow-twitch fibers is well established, data on the fiber type-specific intrinsic mitochondrial function and morphology are highly limited with existing data mainly being generated in animal models. This highlights the need for more human data on the topic.

**Methods:**

Here, we utilized THRIFTY, a rapid fiber type identification protocol to detect, sort, and pool fast- and slow-twitch fibers within 6 h of muscle biopsy sampling. Respiration of permeabilized fast- and slow-twitch fiber pools was then analyzed with high-resolution respirometry. Using standardized western blot procedures, muscle fiber pools were subsequently analyzed for control proteins and key proteins related to respiratory capacity.

**Results:**

Maximal complex I+II respiration was 25% higher in human slow-twitch fibers compared to fast-twitch fibers. However, per mitochondrial volume, the respiratory rate of mitochondria in fast-twitch fibers was approximately 50% higher for complex I+II, which was primarily mediated through elevated complex II respiration. Furthermore, the abundance of complex II protein and proteins regulating cristae structure were disproportionally elevated in mitochondria of the fast-twitch fibers. The difference in intrinsic respiratory rate was not reflected in fatty acid–or complex I respiration.

**Conclusion:**

Mitochondria of human fast-twitch muscle fibers compensate for their lack of volume by substantially elevating intrinsic respiratory rate through increased reliance on complex II.

## Highlights

• Human slow-twitch muscle fibers possess greater respiratory capacity than fast-twitch fibers
• Fast-twitch muscle fibers have lower mitochondrial density than slow-twitch fibers
• Mitochondria in fast-twitch fibers compensate for their low volume by higher intrinsic respiratory capacity
• The higher intrinsic mitochondrial capacity in fast-twitch fibers is driven by faster succinate metabolism
• Mitochondria of the fast-twitch muscle express greater levels of proteins controlling cristae structure

## 1 Introduction

Human skeletal muscle differentiates into two major fiber types based on their myosin heavy chain content, each with highly specialized contractile and metabolic profiles. Slow-twitch type I fibers have a slow peak shortening velocity, a low recruitment threshold, and a reliance on oxidative metabolism due to their high mitochondrial content. In contrast, fast-twitch type II fibers have faster shortening velocity and a higher recruitment threshold. However, they fatigue more easily in part due to protein allocation favoring glycolytic enzymes over mitochondrial content to enable higher catalytic capacity [1,2].

Investigations on the mitochondria of the oxidative and glycolytic fibers in mostly animal models have revealed some differences in intrinsic fat oxidation, reactive oxygen species (ROS) production, and mitochondrial calcium handling [3]. Subsequent work by Mishra et al. [4] showed a more fused mitochondrial network in oxidative (IIA) versus glycolytic (IIX/IIB) fibers. This was followed by Bleck et al. [5] who used 3D renderings to show large differences in mitochondrial network connectivity, intermyofibrillar location, and linked muscle fiber types to inter-mitochondrial junction morphology, which was previously suggested to alter mitochondrial cristae density [6].

Most of the data on fiber type-specific mitochondrial form or function are generated in animal models comparing oxidative to glycolytic muscle fibers [4,5,7]. Type IIA fibers generally tend to fall under the oxidative category together with type I fibers in animal models of metabolism, whereas investigations in human muscle, by contrast, more often categorize muscle according to their twitch speed, thus separating type I from type IIA and IIX, i.e. slow-, versus fast-twitch fibers [[8], [9], [10]]. This makes a translation from animal models to human skeletal muscle difficult as humans lack the glycolytic type IIB fibers and the population of intermediate type IIX fibers is highly limited [11,12]. Adding further complexity to the translation, when compared, complex II levels are significantly greater in type IIA fibers compared to type I in rat hindlimbs [13,14], with respiration showing similar tendencies [15]. Thus, inferring large differences found between oxidative and glycolytic muscle fibers in animals onto human slow- and fast-twitch fibers may be inappropriate. Unfortunately, due to the laborious nature of single muscle fiber analysis, fiber type-specific findings in animal models are seldom further investigated in human muscle.

Recent analysis of the 3D characteristics of the mitochondrial network within human skeletal muscle has indicated two distinct morphologies of intermyofibrillar mitochondria: a high-volume, elongated network mostly aligned parallel to the myofibrils, and a low-volume, fragmented transverse network [16]. We suspect these two mitochondrial populations are a consequence of the different metabolic demands of the two main fiber types. To investigate this, we performed fiber type-specific single muscle fiber analysis on intrinsic molecular markers regulating mitochondrial structure-, and ultrastructure as well as oxidative phosphorylation (OXPHOS) enzyme content. Furthermore, using the novel THRIFTY technique developed in our lab [17,18], we were able to rapidly identify fiber types in individually dissected muscle fibers and for the first time, assess mitochondrial respiration in fiber-type specific pools from human skeletal muscle. This approach coupled with single fiber analysis of molecular markers allowed us to provide novel insights into muscle fiber type-specific mitochondrial function in human skeletal muscle.

## 2 Methods

### 2.1 Ethics statement

This study was conducted in accordance with the declaration of Helsinki. The study was approved by the regional ethics committee in Stockholm (Dnr: 2017/2034-31/2 and Dnr: 2021-00364). All participants were volunteers who gave their written and oral consent prior to enrolment.

### 2.2 Participant characteristics and included muscle fibers

Seven healthy participants were recruited for the study, four women and three men (age 28 ± 3 years, height 171 ± 8 cm, BMI 24 ± 1.7 kg/m^2^, and VO_2_max 45 ± 4 ml/kg/min). A median of 48 (range 15–52) slow-twitch (type I) and 31 (21–54) fast-twitch (type II) fibers were included per participant in the respiratory measurement following fiber typing, with 285 slow-twitch fibers and 232 fast-twitch fibers included across all participants.

### 2.3 Muscle biopsies

All muscle biopsies were collected in a fasted state between 9- and 12 am. Biopsies were taken from the vastus lateralis muscle under local anesthesia (2 % Carbocain, AstraZeneca, Södertälje, Sweden) using a 5 mm Bergström needle with manually applied suction [19]. Each sample was immediately blotted free of excess blood and divided into two pieces, one quickly snap-frozen in liquid nitrogen, freeze-dried, and processed as described under the section *Single muscle fiber analysis*. The second half of the muscle biopsy was processed as described below under the sections *Muscle fiber typing* and *Fiber type-specific respirometry*.

### 2.4 Muscle fiber typing

Immediately following biopsy sampling, the muscle sample was placed in a petri dish filled with ice-cooled biopsy preservation media (BIOPS; 10 mM Ca-EGTA, 0.1 μM free Ca, 20 mM imidazole, 20 mM taurine, 50 mM K-MES, 0.5 mM Dithiothreitol, 6.56 MgCl_2_, 5.77 mM ATP, 15 mM phosphocreatine, pH 7.1). The petri dish was placed on a bed of ice under a stereomicroscope and 80 individual muscle fibers were dissected out of the sample and placed in individual droplets of BIOPS allocated in a gridded plastic container. The dissected muscle fibers were fiber typed according to our recently developed THRIFTY protocol [17,18]. Briefly, an end of each muscle fiber (≈0.5 mm) was cut off and placed on a microscope slide pre-printed with a coordinate grid system. Fiber ends were stained for myosin heavy chain (MHC) I & II (BA-F8, SC-71; specifications for solutions in Supplementary Table 1) and identification was performed in a fluorescent microscope. The remaining sections of the fibers were subsequently pooled according to their fiber type. The whole procedure, from biopsy sampling to typed and pooled fibers, took approximately 5–6 h, during which fibers were constantly kept in ice-cooled BIOPS, which is sufficiently fast to not significantly affect respiratory output of the fibers [20].

### 2.5 Fiber type-specific respirometry

The slow- and fast-twitch muscle fiber pools were permeabilized for 15 min in saponin solution (50 μg/1 ml BIOPS) and subsequently washed in BIOPS before measurement. Mitochondrial respiration and H_2_O_2_ emission were measured using a two-channel high-resolution respirometer with an attached fluorescent probe (Oxygraph-2k, Oroboros Instruments Corporation, Innsbruck, Austria). Pools of muscle fibers were placed into the 2 ml wells containing respiration medium MIR05 (EGTA 0.5 mM, MgCl_2_6H_2_O 3 mM, K-lactobionate 60 mM, Taurine 20 mM, KH_2_PO_4_ 10 mM, HEPES 20 mM, Sucrose 110 mM, and BSA 1g L^−1^). The type I and & II fiber pools were loaded onto different chambers in a randomized order and the same protocol was carried out simultaneously in both chambers; 0.2 mM octanoyl carnitine + 0.5 mM malate (fat leak), 2.5 mM ADP (fat respiration), 10 mM glutamate + 5 mM pyruvate (Complex I), 10 mM succinate (Complex I + II), 0.5 μM rotenone (Complex II), 10 μM cytochrome C (membrane integrity), and 0.05 μM FCCP titration (uncoupled respiration). All experiments were performed at 37 °C and prior O_2_ calibration was completed per the manufacturers' instructions. Following respirometry, the entirety of the 2 ml chambers was collected to determine protein loading control and to measure specific proteins). The wells were rinsed in dH_2_O approximately ten times to make sure all muscle fibers were collected. The collected material was stored in falcon tubes at −80 °C until further analysis. All respiratory data collected are related to calculated wet weight based on Pan actin immunostaining or mitochondrial content calculated from VDAC1+2, TOMM20, CIII & CIV immunostaining, as described below. All measurements and analyses were performed in DatLab 5.2 software (Oroboros, Paar, Graz, Austria). The addition of cytochrome c increased respiration by 5- to 8 % in type I and type II fibers (p < 0.05), indicating only minor damage to the mitochondrial membrane. No difference in the increase in respiratory rates between the fiber types was observed following cytochrome c addition.

### 2.6 Homogenization of fiber pools

Fiber samples collected from the wells of the Oxygraph-2k were centrifuged at 4 °C for 10 min at 3 000 g. Most of the supernatant was removed from the sample, and approximately 1 ml was left in each of the falcon tubes. The 1 ml solution was transferred into a microtube and spun at 4 °C for 15 min at 16 000 g. The supernatant was carefully removed, and the pelleted fibers were rinsed in 500 μl of dH2O. The centrifugation and washing procedure were repeated once. ZrO 0.5 mm beads were then added to the sample, followed by 200 μl of ice-cooled homogenization buffer (2 mM HEPES pH 7.4, 1 mM EDTA, 5 mM EGTA, 10 mM MgCl_2_, 50 mM B-glycerophosphate, 1 % Triton X, 1 % Phosphatase Inhibitor Cocktail 3 (Sigma–Aldrich P0044) and 1 % Halt Protease Inhibitor Cocktail (Thermo Scientific, Rockford IL)). The samples were immediately processed in a BulletBlender (NextAdvance, Averill Park, NY) until completely homogenized. Samples were then left to shake for 30 min and subsequently rotated for an additional 30 min. Homogenization, shaking, and rotation was carried out in a cold room at 4 °C. Samples were then diluted in 4× Laemmli sample buffer and denatured at 95 °C for 5 min before storage at −30 °C until immunoblotting.

### 2.7 Calibration curve for calculation of wet weight and protein quantification

To calculate the wet weight of each fiber pool, a calibration curve was prepared from freeze-dried muscle samples from five participants. The samples were manually dissected free from blood and connective tissue under a stereo microscope, pooled and subsequently weighed on an ultra-micro balance with readability of 0.1 μg (Cubis® MCA2.7S – 2S00 – M, Sartorius Lab Instruments GmbH & Co, Göttingen, Germany). The pooled sample was homogenized in 1 μl buffer per 1 μg sample according to the protocol described below and serially diluted five times. The calibration curve was loaded onto each gel together with the pooled fiber samples recovered from the respirometry chambers. The calibration curve yielded near-perfect linearity for actin immunostaining (R^2^ = 0.9999–0.9997, Supplemental Figure 1). Thus, the actin immunostaining from each sample was used to calculate the sample wet weight [21]. A dry-to-wet weight ratio of ¼ was used for all samples.

### 2.8 Immunoblotting of fiber pools

**Figure 1:**
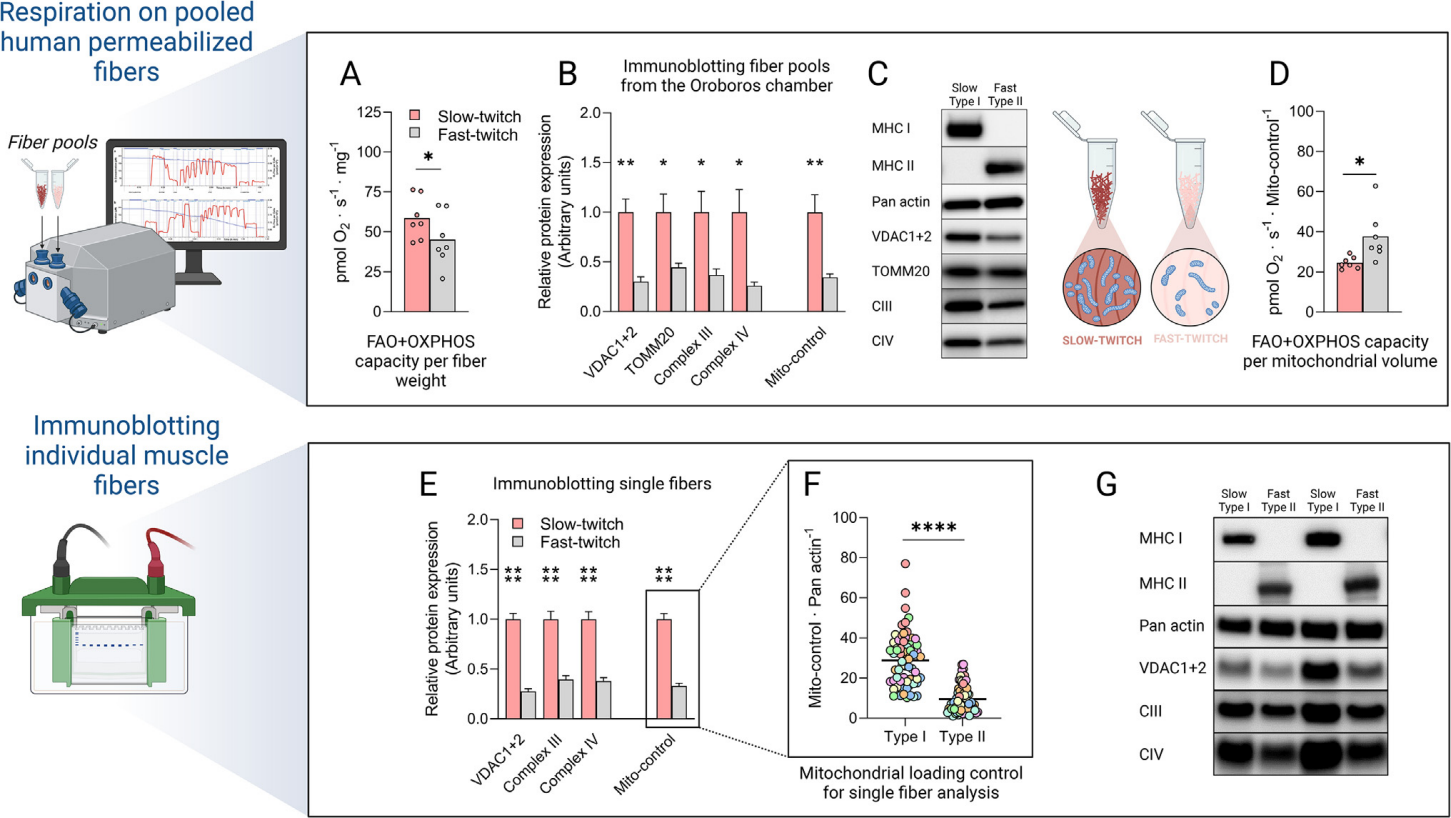
**Respiration and mitochondrial content in human slow- and fast-twitch muscle fibers**. **A**, FAO + OXPHOS respiratory capacity in pools of permeabilized slow- (type I) and fast-twitch (type II) human muscle fibers. A total of 285 slow and 232 fast-twitch fibers were included. Data represent the mean and individual values of seven participants. ∗p < 0.05 (Paired samples t-test). **B**, Expression of mitochondrial loading control proteins separately related to Pan actin and averaged into a mitochondrial loading control value (Mito-control) in the pools of permeabilized slow- and fast-twitch fibers used for respiratory measurements ∗∗p < 0.01 (Paired samples t-test). **C,** Representative blots from the data presented in Figure 1B from one subject. **D,** FAO + OXPHOS respiratory capacity per mitochondrial volume (Mito-control) in pools of permeabilized slow- and fast-twitch human muscle fibers. The number of fibers, subjects, and statistical analysis are the same as in Figure 1A. **E,** Mitochondrial loading control proteins related to Pan actin and an averaged Mito-control in 69 slow vs. 70 fast-twitch single muscle fibers from the same seven participants as Figure 1A–D. ∗∗∗∗p < 1 · 10^−5^ (Mann–Whitney U). **F,** Mito-control presented on individual fiber level in 69 slow vs. 70 fast-twitch single muscle fibers from the same seven participants as Figure 1A–D. Colors indicate fibers from different participants; lines indicate mean values. **G,** Representative blots from the data presented in Figure 1E,F. Blots corresponding to the subject presented in green. FAO = Fatty acid oxidation, OXPHOS = Oxidative phosphorylation.

Together with the calibration curve, samples were loaded onto 18-well Criterion TGX gradient gels (4–20% acrylamide or AnykD; BioRad). Electrophoresis was performed for 28 min at 300 V in electrophoresis buffer (25 mM Tris base, 192 mM glycine, and 3.5 mM SDS) on a bed of ice in a cold room at 4 °C. The gels were then equilibrated in transfer buffer (25 mM Tris base, 192 mM glycine, and 10% methanol) for 30 min at 4 °C. Transfer of proteins to polyvinylidene difluoride membranes (BioRad) was carried out at 300 mA for 3 h at 4 °C. Membranes were stained with MemCode Reversible Protein Stain Kit (Thermo Scientific) to confirm successful transfer and to aid in sectioning the membrane before primary antibody incubation. After membrane sectioning and destaining, blocking was carried out for 1 h using 5 % milk in Tris-buffered saline with Tween (TBST; 20 mM Tris base, 137 mM NaCl, and 0.1% tween). Membranes were subsequently washed 3 × 3 min in TBST prior to overnight incubations with primary antibodies. Membranes were stained for MHC I and MHC II to check fiber pool purity, Pan actin for calculation of wet weight and VDAC1+2, TOMM20, CIII and CIV for mitochondrial volume (Figure 1C). Antibody information is presented in Supplemental Table 1. Following overnight incubation, membranes were rewashed 3 × 5 min in TBST, followed by 1 h of secondary antibody incubation at room temperature. Again, membranes were washed 3 × 5 min in TBST and then finally incubated in SuperSignal West Femto Maximum Sensitivity Substrate (Thermo Scientific) before visualization in a ChemiDoc MP with Quantity One software (Version 6.0.1; Biorad). Blots were quantified in Image Lab (Version 6.0.1; Biorad).

### 2.9 Calculation of mitochondrial loading control

The mito-control for respiratory measurements (Figure 1B,C) were calculated as an average of proteins VDAC1+2, TOMM20, CIII and CIV (antibodies specified in Supplemental Table 1) obtained during western blotting. These staining's were done on the same fibers that were used in the Oroboros O2k chamber. A ratio between slow- and fast twitch fiber expression of each protein, where slow-twitch fiber expression was set to 1 (Figure 1C) was initially created. Next, an average ratio of the four proteins for each volunteer was calculated and used as mitochondrial loading control for the respiratory rates. Likewise, for the single muscle fiber western blots on lyophilized muscle fibers, a mito-control was created for each individual fiber as an average of VDAC1+2, CIII and CIV. TOMM20 was not stained for on these fibers since both CI (22 kDa), FIS1 (17 kDa), and SOD2 (22 kDa) were prioritized on the PVDF-membrane of each fiber, making an additional TOMM20 staining (16 kDa) difficult.

While we recognize that choosing protein markers to match mitochondrial volume across different cell types may be difficult, we had a couple of considerations in mind prior to choosing staining protocol for the western blot. First and foremost, we wanted membrane bound mitochondrial markers, as we were uncertain of how running respiratory measurements as well as homogenization of fibers would affect non-membrane bound protein leakage from the mitochondrial matrix. Since the fibers recovered from the respiratory chamber were incubated in MIR05 containing BSA, the fibers had to be carefully washed so that the high concentration of BSA would not interfere with the subsequent western blot procedures, thus risking some non-membrane proteins to be washed from the sample.

To increase the reliability of the data, an average of four (fibers from respirometry chamber) or three (single fiber analysis) membrane bound proteins were chosen as loading control. Proteins located on both the inner and outer mitochondrial membrane were chosen to account for variations in mitochondrial outer membrane volume and cristae density. Proteins within CIII and CIV were chosen as they linearly couple the redox reaction from CoQ regardless of where electrons enter the ETC. Thus, CIII and CIV should be dimensioned evenly within mitochondria due to their co-dependance during respiration. Moreover, both CIII and CIV have been shown to be fair predictors of mitochondrial volume and cristae area (r ≈ 0.6, respectively) [22]. As for protein markers of mitochondrial volume on the outer mitochondrial membrane, a myriad of previously published papers use either TOMM20 or VDAC1+2 as markers for OMM quantity or mitochondrial volume. Both markers have also been shown to parallel total mitochondrial protein levels [23]. Thus, we choose these proteins to keep the mitochondrial loading control somewhat consistent with a large portion of the literature that utilizes the content of a specific protein as a mitochondrial marker. Importantly, all proteins showed similar patterns of expression which lends further confidence to an accurate estimation of mitochondrial content in the two fiber types.

### 2.10 Single muscle fiber western blot

Freeze-dried samples were manually dissected under a stereomicroscope. Approximately 50–100 fibers were dissected from each biopsy sample and subsequently typed according to the THRIFTY protocol [17,18]. Ten fibers of each type from every volunteer were homogenized individually in 20 μl of ice-cooled homogenization buffer with 1× Laemmli sample buffer. The entirety of each sample was then loaded onto each well of 26-well Criterion TGX gradient gels (AnykD; BioRad), separated and transferred to PVDF membranes as described above. After blocking, antibody incubation and visualization, membranes were stripped using Restore PLUS Western Blot Stripping Buffer (Thermo Scientific) for 45 min at room temperature for staining of additional protein targets. All antibody information is compiled in Supplemental Table 1. One type I fiber was excluded from analysis due to a slight MHC II cross-contamination. The first fully analyzed volunteer was not stained for OPA1 and MIC60. One participant also had MFN2, MIC60, and FIS1 stained on separate fibers compared to the rest of the markers. For these fibers, control proteins (MHC I, MHC II, Pan actin and VDAC 1 + 2) were stained, but not CIII and CIV. Thus, based on the previous 20 fibers from this volunteer, a control ratio was used to estimate Mito-control based on VDAC1+2 staining alone.

### 2.11 Statistics

Values are presented as mean ± SD for respirometry data and as individual values for single fiber data. Normality was tested with Shapiro–Wilks. Respirometry data were analyzed using paired samples t-test and single fiber data were analyzed with a Mann–Whitney U test. Correlations were calculated with Pearson correlation coefficient.

## 3 Results

### 3.1 Total- and intrinsic mitochondrial respiratory capacity in human skeletal muscle fiber types

**Figure 2:**
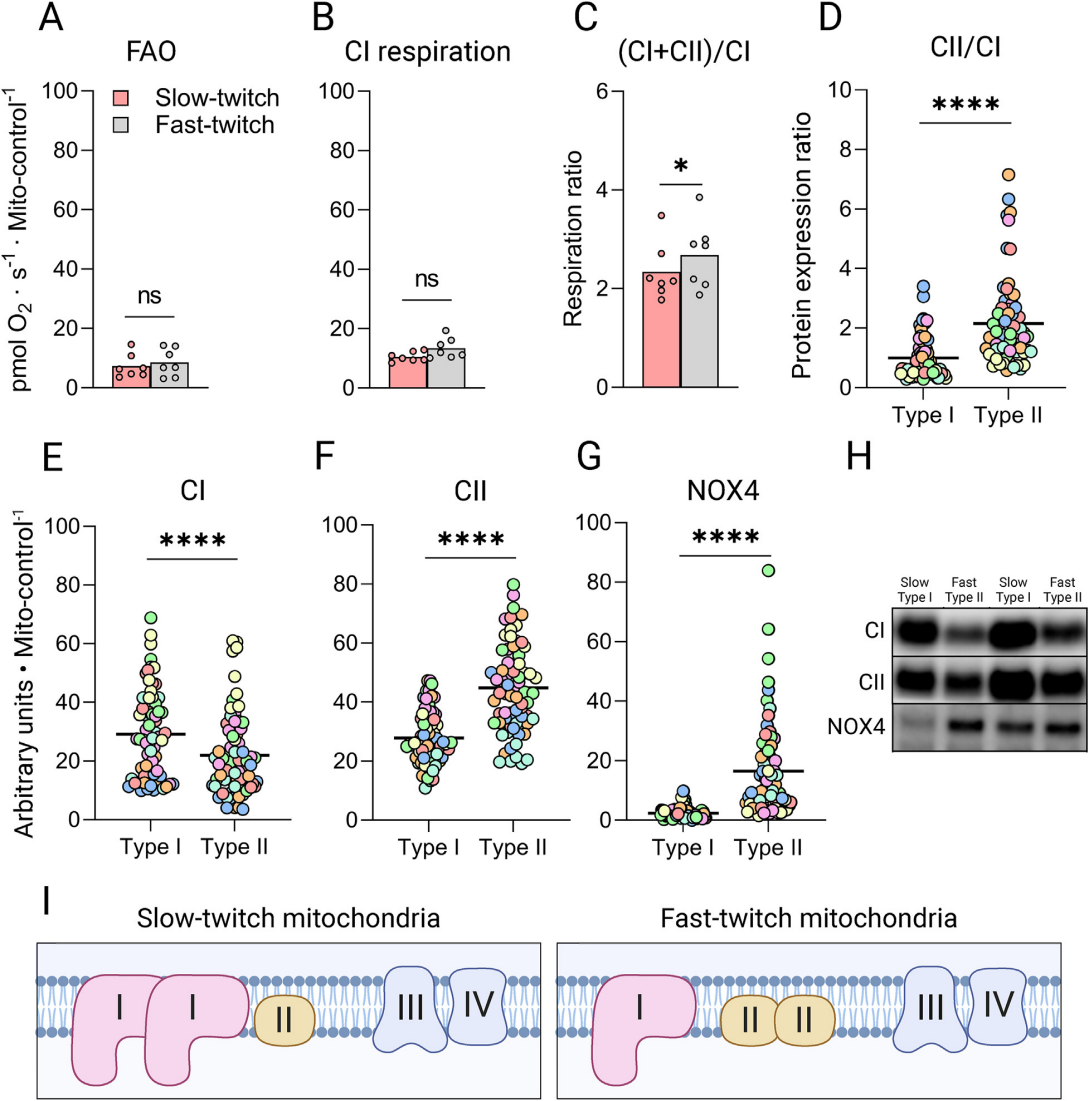
**Electron entry points of theIMMin human skeletal muscle fiber types. A & B,** FAO and CI respiration related to mito-control in pools of permeabilized slow- and fast-twitch human muscle fibers. A total of 285 slow and 232 fast-twitch fibers were included. **C,** OXPHOS capacity (CI + CII respiration) related to CI respiration. Data represent the mean and individual values of seven participants. ∗ = p < 0.05, ns = not significant (Paired samples t-test). **D,** Protein expression ratios of CI and CII within type I and type II fibers ∗∗∗∗p < 1 · 10^−5^ (Mann–Whitney U). **E-G,** Protein expression of CI, CII & NOX4 in 69 slow vs. 70 fast-twitch fibers from seven participants. Colors indicate fibers from different participants; lines indicate mean values. ∗∗∗∗p < 1 · 10^−5^ (Mann–Whitney U). **H,** Representative blots for Figure 2D–F. Blots corresponding to the subject presented in green. Mito-control values and blots are presented in Figure 1E–G. **I**, Graphical interpretation of C-F. IMM = Inner mitochondrial membrane, FAO = Fatty acid oxidation, CI = Complex I, CII = Complex II, OXPHOS = Oxidative phosphorylation.

**Figure 3:**
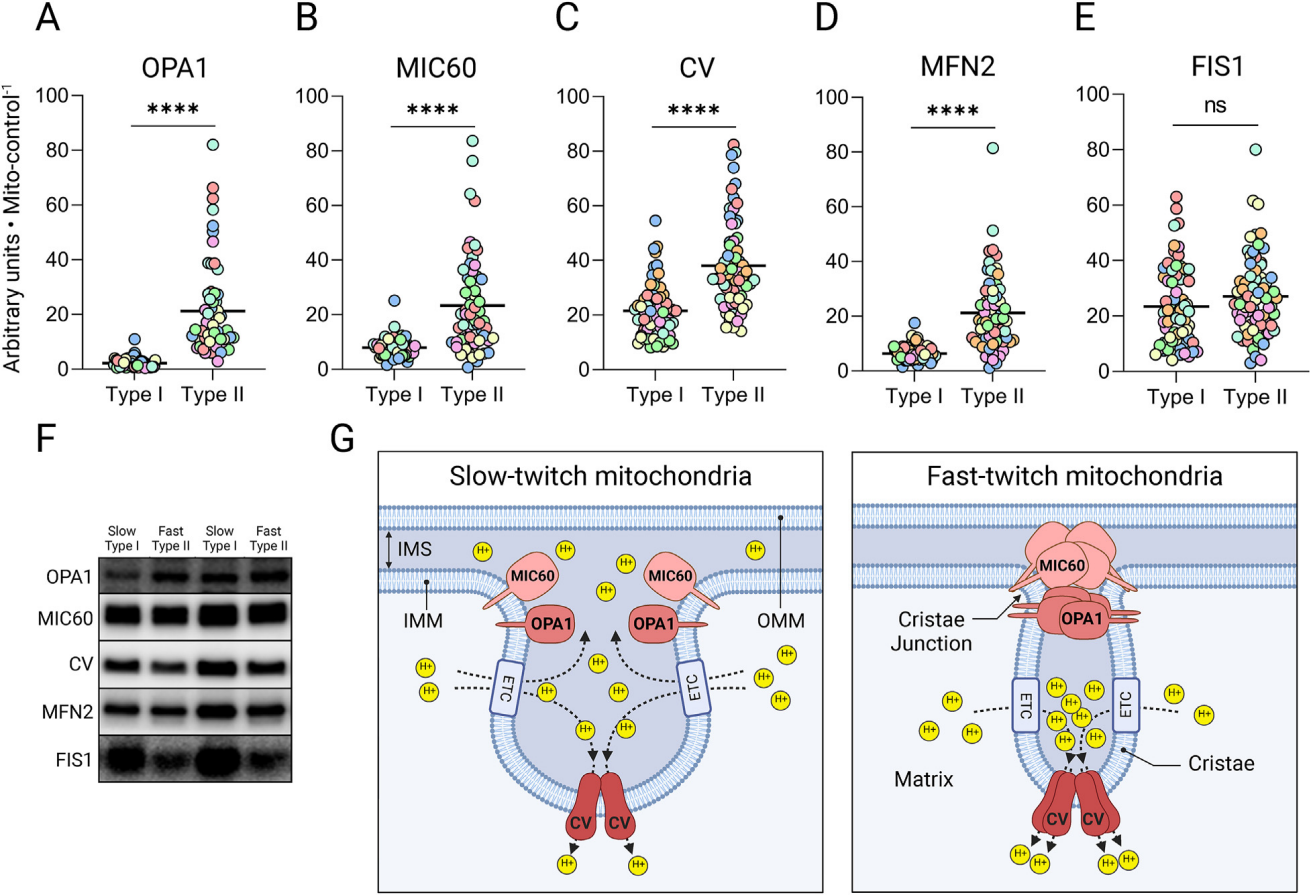
**Mitochondrial ultrastructure and morphology in human skeletal muscle fiber types. A-E**, Protein expression related to mitochondrial loading control. OPA1 and MIC60 in 59 slow vs. 60 fast-twitch fibers from six participants (**A & B**), and CV, MFN2 and FIS1 expression in 69 slow vs. 70 fast-twitch fibers from seven participants (**C-E**). Colors indicate fibers from different participants; lines indicate mean values. ∗∗∗∗p < 1 · 10^−5^ (Mann–Whitney U). **F**, Representative blots from four single muscle fibers corresponding to the participant represented in green. Mito-control values and blots are presented in Figure 1E–G, **G**, Graphical interpretation of A-C. CV = Complex V; ATP-synthase.

Previous investigations on mitochondrial respiratory properties in the different muscle fiber types have all used various animal models comparing muscle predominantly expressing oxidative or glycolytic fibers [3,24]. Here, we succeeded in measuring respiration in completely pure pools of permeabilized human slow- and fast-twitch muscle fibers in seven healthy volunteers. As expected, full ADP stimulated respiration, i.e. fatty acid oxidation (FAO) plus OXPHOS capacity (substrate; octanoyl carnitine, malate, glutamate, pyruvate, and succinate) was higher in the slow-twitch fibers at 59 pmol x s^−1^ x mg^−1^ compared to the observed respiratory rate of 47 pmol x s^−1^ x mg^−1^ in the fast-twitch fibers, Figure 1A. Following the high resolution respirometry in the Oxygraph, the fibers analyzed in the respiratory chamber were recovered and analyzed for mitochondrial volume control proteins (henceforth referred to as mito-control; described in more detail in the methods section). The mito-control proteins include proteins on the outer- (voltage-dependent anion channel 1 & 2; VDAC1+2, translocase of the outer mitochondrial membrane complex subunit 20; TOMM20) and inner mitochondrial membrane (ubiquinol-cytochrome C oxidoreductase; CIII, cytochrome C oxidase; CIV). These proteins jointly indicated an approximately three-fold higher mitochondrial volume in the slow-twitch fibers (Figure 1B,C). This large difference in mitochondrial volume is consistent with a previous report using focused ion beam-scanning electron microscopy [16] in humans, however, others before that have indicated smaller fiber type differences with approximately 60% higher mitochondrial volume in slow-twitch fibers using conventional electron microscopy [25]. Three of the mito-control proteins (VDAC1+2, CIII, & CIV) were again stained for during subsequent immunoblotting of lyophilized single muscle fibers from the same volunteers (Figure 1E–G) to obtain a mitochondrial loading control from single fiber protein analysis (Data presented in Figure 2, Figure 3). The lyophilized fibers revealed a similar pattern of approximately three-fold higher mito-control in the slow-twitch fibers (Figure 1E–G) confirming the difference in mitochondrial content in the fibers recovered from the respiratory chambers. Next, utilizing the mito-control presented in Figure 1B, we investigated the FAO + OXPHOS capacity per mitochondrial volume in the two fiber types. This revealed a significantly greater maximal respiratory rate in the mitochondria of fast-twitch fibers compared to in slow-twitch fibers (Figure 1D).

### 3.2 Electron entry points to the inner mitochondrial membrane

We discovered a 50% higher capacity for intrinsic FAO and OXPHOS in the fast-twitch fiber mitochondria (Figure 1D). This finding contrasts prior work in animal models showing similar [24] or higher [7] mitochondrial capacity in smaller mammals' red muscle tissue. An explanation for the discrepancy between our finding to that of the literature in animals may be that oxidative ‘red’ muscle in animals often includes a mixture of type IIA and type I fibers, whereas type IIA and type I fibers in this paper, and much of the human literature, are on opposing ends of the comparisons. During our analysis, no difference in the respiratory rates was however observed during states of FAO (substrate; octanoyl carnitine and malate) and complex I capacity (FAO substrates plus glutamate and pyruvate) between the mitochondria of the slow- and fast-twitch fibers (Figure 2A,B). This suggests that the higher FAO + OXPHOS capacity in the fast-twitch fibers is primarily due to an elevated capability to metabolize succinate (Figure 2C). As for protein expression, the two dehydrogenases of the electron transport chain (ETC), NADH dehydrogenase; complex I (CI), and succinate dehydrogenase; complex II (CII), were differentially expressed in the two fiber types when related both to our mito-control (Figure 2D–F) as well as when related to the ETC's electron demanding CIII & CIV protein complexes (data not shown). In reference to the existing literature, the higher CII/CI protein ratio in the fast-twitch fibers is partly reflected in one [26], but not both [2] proteomics data sets currently available in fast- and slow-twitch fibers of young individuals.

The levels of NADPH oxidase 4 (NOX4) were nearly ten-fold higher in fast-twitch as compared to slow-twitch fibers related to their respective mitochondrial content (Figure 2G) and approximately 40% higher in relation to their respective fiber size (Supplemental Figure 1J). This may suggest that the fast-twitch fibers have an additional route to alleviate NADH oxidation pressure onto CI. Partly located at the IMM and the sarcoplasmic reticulum in muscle, NOX4 oxidizes NADH and consequently produces O_2_- and H_2_O_2_ [27]. It has recently been suggested that NADH oxidation by NOX4 is the main origin of exercise-induced reactive oxygen species (ROS) in skeletal muscle [28]. As the deletion of NOX4 has been linked to reductions in antioxidant defenses and reduced superoxide dismutase 2 (SOD2) content [28], we also investigated SOD2 levels in the two fiber types. However, despite a much lower NOX4 expression in the slow-twitch fibers, we found SOD2 to be equally dimensioned in the mitochondria of slow- and fast-twitch muscle (Supplemental Figure 3), which could suggest the higher NOX4 in fast-twitch fibers may be reflecting cytosolic, rather than mitochondrial content.

### 3.3 Mitochondrial membrane dynamics and intrinsic respiratory rate

In striking contrast to respiration (Figure 1A), OXPHOS enzyme content, and other mitochondrial proteins on a per fiber basis (Supplemental Figure 2A-L), we found that levels of inner mitochondrial membrane (IMM) fusion and mitochondrial cristae remodeling protein optic atrophy 1 (OPA1) [29] were significantly greater expressed in fast-twitch fibers (Supplemental Figure 2F) despite the three-fold higher mito-control in the slow-twitch fibers (Figure 1E). Related to mitochondrial volume, OPA1 expression in fast-twitch fibers was accentuated even more, with levels 10-fold over that found in mitochondria of slow-twitch fibers (Figure 3A). Likewise, MIC60, a key component in the mitochondrial inner membrane organizing system (MINOS) [30,31], was also expressed in a fiber type-dependent manner with nearly three-fold higher expression in fast-twitch fiber mitochondria (Figure 3B). Additionally, fast-twitch mitochondria expressed double the ATP-synthase (Complex V; CV) levels of mitochondria in slow-twitch fibers (Figure 3C). Though the central function of CV is phosphorylation of ADP, it has additional roles in regulating cristae formation. CV exists as a V-shaped dimeric complex. The V-shaped structure of the hydrophobic F_0_ membrane-embedded body directly imposes a folding of the IMM, forming the cristae rims [32]. Taken together, we observed three proteins that have been reported to work separately [[31], [32], [33]] and synergistically [[34], [35], [36]] to uphold a tight and energetically efficient cristae structure, which are more abundant in fast-twitch mitochondria.

Fast-twitch mitochondria express higher levels of pro-fusion protein mitofusin 2 (MFN2) but not fusion inhibitor protein (mitochondrial fission protein 1; FIS1) (Figure 3D,E) [37,38]. We suspect that the higher MFN2 levels in fast-twitch mitochondria to some extent reflect a larger proportion of mitochondria tethered to the sarcoplasmic reticulum located between the myofibrils [39] to aid in handling the greater cytosolic calcium release seen with contraction in fast-twitch fibers [40]. This notion is also in line with our finding that CV is more abundant in fast-twitch fiber mitochondria, as intermyofibrillar mitochondria have previously been reported to contain more CV and less CIV compared to subsarcolemmal mitochondria [41] (Supplemental Figure 4).

## 4 Discussion

Here we employed a novel technique to measure human muscle fiber type specific mitochondrial respiration, coupled with protein expression analysis of key proteins regulating mitochondrial form and function, to gain insight into mitochondrial specialization in human slow- and fast-twitch muscle fibers. We observed that the mitochondria of the fast-twitch muscle fibers are significantly less abundant, but compensate for their relatively low volume by upregulating crucial proteins regulating mitochondrial bioenergetics and dynamics which ultimately leads to a faster succinate metabolism in the fast-twitch mitochondria. We suspect that a mechanism of elevated CII content in the fast-twitch mitochondria may in part be that the fast-twitch muscle fibers utilize the REDOX regulated bidirectionality of CII [42]. During exhaustive exercise where local hypoxia occurs, especially in the center of the larger and less perfused fast-twitch myofiber, CII may short-circuit the ETC to oxidize the ubiquinol (CoQH_2_) pool when oxygen diffusion to the centrally located mitochondria does not suffice. The fast-twitch fibers would thus need an additionally large pool of CII by the sarcolemma to metabolize both the succinate produced by CII reversal in intermyofibrillar mitochondria and for oxidizing succinate during regular substrate metabolism. The hypothesis that oxidative phosphorylation runs, at least in part, more anaerobically the further from the sarcolemma it is located, has support in both O_2_ diffusion into the myofiber being disproportionately limited past the first few microns from the sarcolemma [43], as well as the mitochondrial CV/CIV ratio increasing with the distance from the sarcolemma [41].

Alternatively, a metabolic state may be occurring at the onset of anaerobic respiration, where the fast- and slow-twitch fibers exchange lactate for succinate to favor their respective ETC composition (Figure 2C–F). As lactate yields additional NADH from complete oxidation compared to pyruvate, this may add additional urgency on CI to reduce the ubiquinone (CoQ) pool to CoQH_2_ prior to CII involvement. Depending on the severity of the CoQH_2_ REDOX pressure within the IMM of the slow-twitch fibers, CII may either halt succinate oxidation or even reverse CII to produce succinate and alleviate some of the CoQH_2_ pressure [42]. In either case, succinate levels will subsequently accumulate and get released into the interstitium of the muscle [44]. The succinate from slow-twitch fibers could then act as a substrate for fast-twitch fibers tailored towards succinate oxidation. Thus, fast- and slow-twitch fibers might exchange lactate and succinate during heavy exercise to symbiotically favor their respective predisposition towards CI or CII respiration.

Regardless of whether succinate is utilized as an electron carrier between intermyofibrillar and subsarcolemmal mitochondria within fast-twitch fibers, or shuttled between fiber types, both would indicate a metabolic strategy that generates ATP faster, but at the cost of a reduced O_2_ efficiency once fast-twitch fibers are recruited. Such a mechanism would fit the previously suggested metabolic state of an increased bypass of CI during heavy exercise to prioritize catalytic speed over substrate efficiency prior to any substantial lactate accumulation [45]. Moreover, our results of increased CII-respiration in the fast-twitch fibers may explain the increased O_2_ cost per produced watt during heavy aerobic exercise, causing a non-linear relationship in O_2_ consumption during the final minutes of gradient exercise tests to exhaustion [46].

Protein expression of OPA1, MIC60, and CV, all synergistically orchestrating cristae folding, were significantly more abundant in fast twitch mitochondria (Figure 3A–C). A tighter crista folding has been associated with an increased respirasome assembly [33] and, consequently, an elevated respiratory rate [33,47] which may in part explain the unexpected finding that the mitochondria of fast-twitch muscle fibers have an approximately 50% higher intrinsic respiratory rate (Figure 1D). Additionally, less respirasome formation due to a slower cristae structure regulation may also explain why the slow-twitch fibers could not translate the higher CI protein expression (Figure. 2D) into an elevated capacity for oxidation of NADH through CI (Figure 2B). Moreover, the OPA1 and MINOS-dependent constriction of the cristae junction is specifically suggested to uphold membrane potential over the IMM by preventing proton leakage into the intermembrane space, thus driving proton re-entry to the matrix faster through CV [29,36], possibly affecting the P/O ratio of the different mitochondria (Figure 3G).

A greater cristae density may be an obvious explanation as to why many of the cristae-upholding proteins are more highly expressed in fast-twitch mitochondria. As space within the muscle cell is a scarce commodity, whereby fast-twitch fibers prioritize organelles related to contraction over mitochondria [48], cristae density, rather than mitochondrial volume, may be the preferred adaptation to increase muscle oxidative capacity in the fast-twitch fibers specifically. However, the limited available data on fiber type-specific cristae density in human muscle (using z-line width to identify muscle fiber type) does not support this notion [49].

Since we find both OMM fusion factors (MFN2; Figure 3D) and IMM fusion- and cristae modulating factors (OPA1 & MIC60; Figure 3A,B) to be more highly expressed in fast-twitch mitochondria, we suspect this is a compensatory mechanism by which aerobic ATP-production can be rapidly upregulated in the fast-twitch fibers despite their lower mitochondrial abundance compared to slow-twitch fibers. As mitochondria are more abundant in slow-twitch fibers, it should more easily uphold a larger fused grid-like structure [4,16]. A smaller pool of mitochondria, might more readily rely on MFN2 and other OMM fusion proteins to initiate rapid morphological changes upon fast-twitch fiber recruitment during exercise. This may imply either inducing transient mitochondrial membrane interactions without initiating full mitochondrial fusion [50], which highly influences mitochondrial cristae morphology [6], or, the formation of nanotunnels [51] to increase respiratory efficiency [52].

In conclusion, human fast- and slow-twitch muscle fibers host distinct mitochondrial populations. Slow-twitch mitochondria favor sheer mitochondrial volume and rely on CI for efficient oxidative coupling and NADH oxidation. By contrast, fast-twitch mitochondria are tailored for speed rather than efficiency through faster succinate metabolism and significantly elevated CII expression. The fast-twitch mitochondrial proteome may enable faster and more efficient mitochondrial morphology changes during high energy demands, likely as a compensatory mechanism for low total volume.

## 5 Limitations of the study

A limitation of the current study is the use of western blot methodology to indirectly assess mitochondrial content. Despite extensive efforts to make this method quantitative, we fully acknowledge that the magnitude of difference in mitochondrial content, as assessed here, is unusually large. While similar differences in human [16] and mouse [5] muscle have been reported previously, a large part of the literature reports considerably smaller differences between the fiber types. It therefore remains to be determined if our assessment using multiple membrane-bound markers reflects actual mitochondrial volume. Given the risk of overestimating the differences in mitochondrial content between type I and type II fibers, the intrinsic difference in fiber type-specific respiration should be interpreted with caution. Thus, to fully confirm the findings presented here, additional studies are required in which intrinsic fiber type-specific respiration is normalized using additional methods of assessing mitochondrial content such as proteomics [53], transmission- [54], or focused ion beam scanning electron microscopy [5,16].

## Funding

This study was funded by project grants awarded to W.A. (P2021-0173, P2022-0022) and F.L. (P2022-0049), respectively, from the 10.13039/501100005350Swedish Research Council for Sport Science. During this work W.A. was also supported by an Early Career Research Fellowship from the 10.13039/501100005350Swedish Research Council for Sport Science (no. D2019-0050). In addition, SE has been awarded project grants from Elisabeth and Gunnar Liljedahls foundation.

## CRediT authorship contribution statement

**Sebastian Edman:** Writing – review & editing, Writing – original draft, Visualization, Validation, Methodology, Investigation, Funding acquisition, Formal analysis, Data curation, Conceptualization. **Mikael Flockhart:** Writing – review & editing, Investigation. **Filip J. Larsen:** Writing – review & editing, Supervision, Investigation, Funding acquisition, Conceptualization. **William Apró:** Writing – review & editing, Supervision, Methodology, Funding acquisition, Conceptualization.

## Declaration of competing interest

The authors declare the following financial interests/personal relationships which may be considered as potential competing interests: Sebastian Edman reports financial support was provided by Elisabeth och Gunnar Liljedahls foundation. William Apro reports financial support was provided by Centrum för idrottsforskning. Filip Larsen reports financial support was provided by Centrum för idrottsforskning.

## Data Availability

Data will be made available on request.

## Appendix A Supplementary data

Supplemental Figure 1 Standard curve of Pan-actin. Standard curve used to relate fiber pools collected from respirometry chambers to a corresponding sample weight (R^2^ = 0.9999). Supplemental Figure 1

Supplemental Figure 2 Mitochondrial protein expression related to loading control in Human Skeletal Muscle Fiber Types. A-E & H-I, Protein expression related to Actin in 69 slow vs. 70 fast-twitch fibers from seven participants. **F-G**, Protein expression related to Actin in 59 slow vs. 60 fast-twitch fibers from six participants. Colors indicate fibers from different participants; lines indicate mean values.∗p < 0.05, ∗∗p < 0.01, ∗∗∗p < 0.001, ∗∗∗∗p < 1 · 10^−5^ (Mann–Whitney U). Representative blots are presented in Figure 1, Figure 2, Figure 3 and Supplemental Figure 3. Supplemental Figure 2

Supplemental Figure 3 Intrinsic mitochondrial SOD2 content in human slow- and fast-twitch muscle fibers. Protein expression of SOD2 related to mito-control in 69 slow vs. 70 fast-twitch fibers from seven participants. Colors indicate fibers from different participants; lines indicate mean values. Ns = not significant (Mann–Whitney U). Representative blots from four single muscle fibers corresponding to the participant represented in green. Control values and blots are presented in Figure 1E–G. Supplemental Figure 3

Supplemental Figure 4 Protein expression ratios of CV and CIV within slow and fast-twitch fibers. Colors indicate fibers from different participants; lines indicate mean values. Muscle fibers from each participant were loaded onto separate gels. ∗∗∗p < 0.001, Mann–Whitney U). Representative blots are presented in Figure 1, Figure 3F. Supplemental Figure 4
