# Supplementary figures and images for: Need for speed: Human fast-twitch mitochondria favor power over efficiency
Source: Mol Metab. 2023 Dec 15;79:101854. doi: 10.1016/j.molmet.2023.101854 (PMC10788296; doi:10.1016/j.molmet.2023.101854)

30  $\mu\text{g}$

15  $\mu\text{g}$

7.5  $\mu\text{g}$

3.75  $\mu\text{g}$

1.875  $\mu\text{g}$

0.9375  $\mu\text{g}$

Pan-Actin

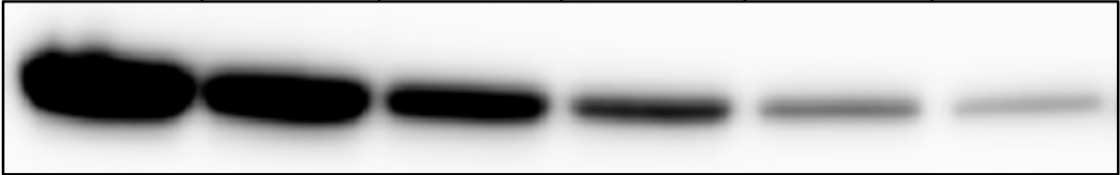

Supplement: Supplemental Figure 1 — Standard curve of Pan-actin. Standard curve used to relate fiber pools collected from respirometry chambers to a corresponding sample weight (R2 = 0.9999). [file mmc1.pdf]

**A****CI**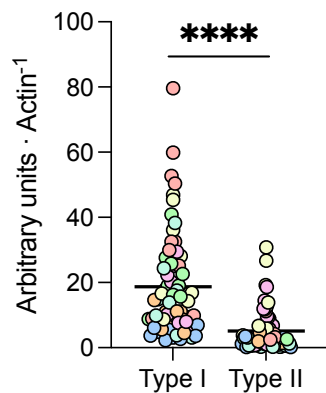**B****CII**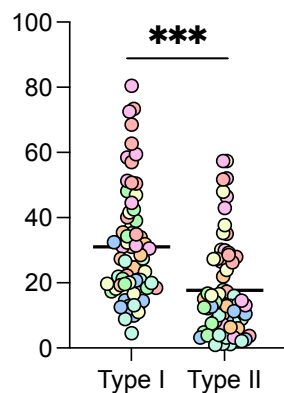**C****CIII**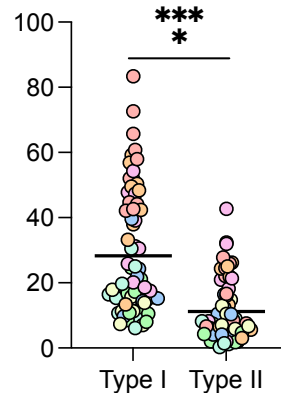**D****CIV**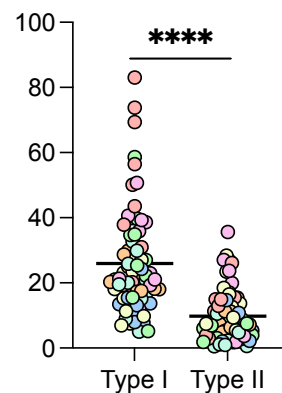**E****CV**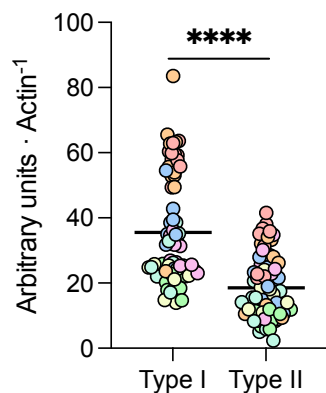**F****OPA1**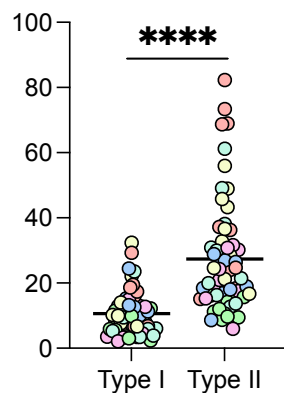**G****MIC60**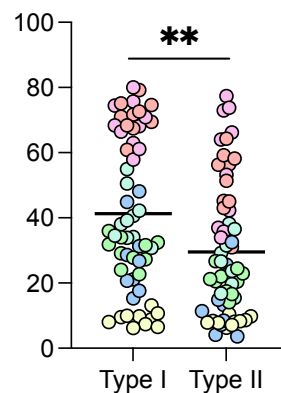**H****MFN2**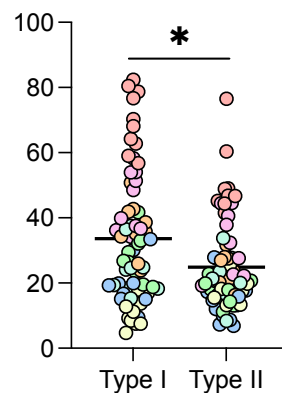**I****FIS1**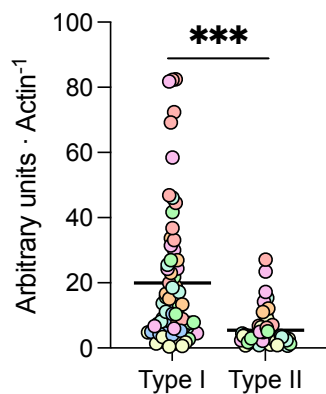**J****NOX4**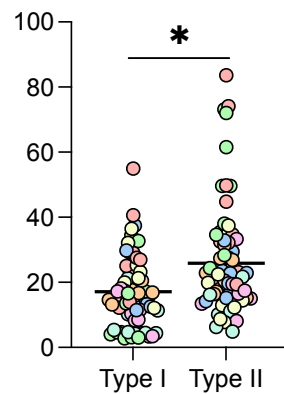**K****SOD2**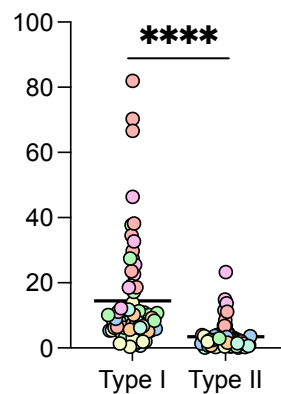**L****VDAC1+2**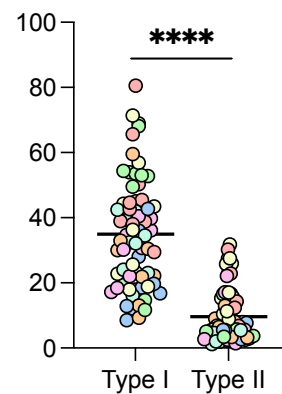

Supplement: Supplemental Figure 2 — Mitochondrial protein expression related to loading control in Human Skeletal Muscle Fiber Types. A-E & H-I, Protein expression related to Actin in 69 slow vs. 70 fast-twitch fibers from seven participants. F-G, Protein expression related to Actin in 59 slow vs. 60 fast-twitch fibers from six participants. Colors indicate fibers from different participants; lines indicate mean values.∗p < 0.05, ∗∗p < 0.01, ∗∗∗p < 0.001, ∗∗∗∗p < 1 · 10−5 (Mann–Whitney U). Representative blots are presented in Figure 1, Figure 2, Figure 3 and Supplemental Figure 3. [file mmc2.pdf]

# SOD2

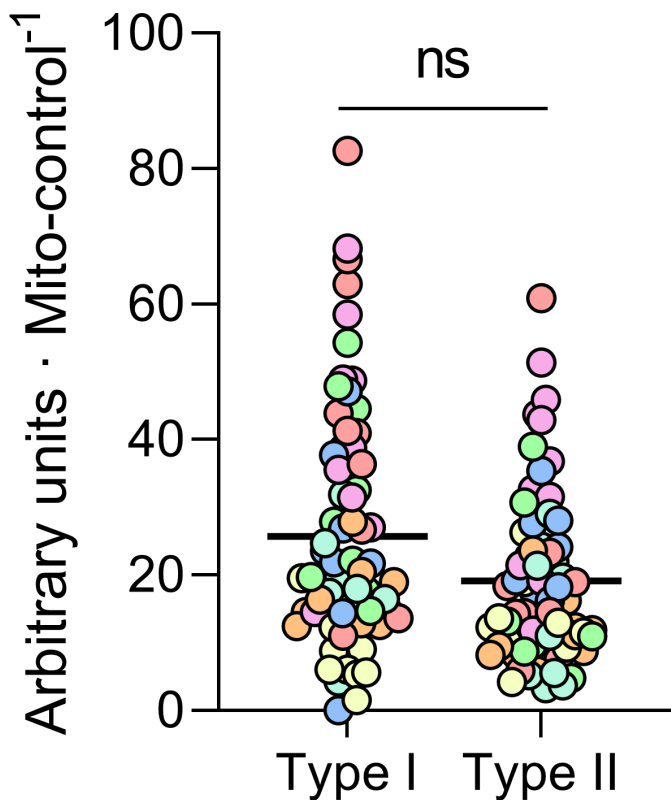

Slow Fast Slow Fast  
Type I Type II Type I Type II

SOD2

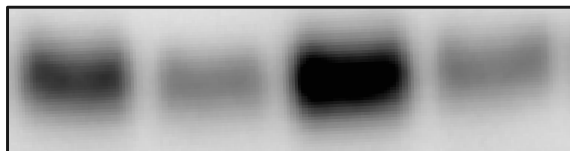

Supplement: Supplemental Figure 3 — Intrinsic mitochondrial SOD2 content in human slow- and fast-twitch muscle fibers. Protein expression of SOD2 related to mito-control in 69 slow vs. 70 fast-twitch fibers from seven participants. Colors indicate fibers from different participants; lines indicate mean values. Ns = not significant (Mann–Whitney U). Representative blots from four single muscle fibers corresponding to the participant represented in green. Control values and blots are presented in Figure 1E–G. [file mmc3.pdf]

**CV/CIV**

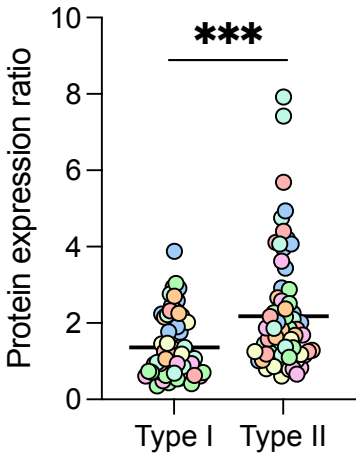

Supplement: Supplemental Figure 4 — Protein expression ratios of CV and CIV within slow and fast-twitch fibers. Colors indicate fibers from different participants; lines indicate mean values. Muscle fibers from each participant were loaded onto separate gels. ∗∗∗p < 0.001, Mann–Whitney U). Representative blots are presented in Figure 1, Figure 3F. [file mmc4.pdf]
