# Supplementary material for: Need for speed: Human fast-twitch mitochondria favor power over efficiency
Source: Mol Metab. 2023 Dec 15;79:101854. doi: 10.1016/j.molmet.2023.101854 (PMC10788296; doi:10.1016/j.molmet.2023.101854)
Supplement: Multimedia component 5 [file mmc5.docx]

**Supplemental Table 1. List of antibodies used with their respective dilution and solution.**

| Target | Source | Identifier | Dilution | Solution |
| --- | --- | --- | --- | --- |
| Pan-Actin | CST | #8456S | 1:20 000 | 2.5 % M-TBST |
| VDAC1/VDAC2 | Abcam | ab154856 | 1:10 000 | 2.5 % M-TBST |
| MHC I | DSHB | BA-F8-s | 1:20 000 | 2.5 % M-TBST |
| MHC II (Fiber pools) | DSHB | SC-71-s | 1:20 000 | 2.5 % M-TBST |
| MHC Anti-fast (Single fibers) | Abcam | Ab91506 | 1:20 000 | 2.5 % M-TBST |
| Total OXPHOS | Abcam | ab110411 | 1:2000 | 5 % M-TBST |
| NDUFB8 | Abcam | ab110242 | 1:10 000 | 5 % M-TBST |
| Mt-CO2 | Proteintech | Cat#55070-1-AP | 1:10 000 | 5 % M-TBST |
| OPA1 | Abcam | ab157457 | 1:500 | 2.5 % M-TBST |
| MFN2 | Abcam | ab56889 | 1:1000 | 2.5 % M-TBST |
| NOX4 | Abcam | ab109225 | 1:1000 | 2.5 % M-TBST |
| SOD2 | Abcam | ab13533 | 1:5000 | 2.5 % M-TBST |
| FIS1 | SCBT | sc-376447 | 1:500 | 2.5 % M-TBST |
| MIC60 (Mitofilin) | SCBT | sc-390707 | 1:500 | 2.5 % M-TBST |
| TOMM20 | Abcam | ab186735 | 1:1000 | 2.5 % M-TBST |
| Anti-mouse IgG HRP-linked | CST | #7076 | 1:10 000, 1:20 000 for MHC I & II | 2.5 % M-TBST |
| Anti-rabbit IgG HRP-linked | CST | #7074 | 1:10 000 | 2.5 % M-TBST |
| MHC I (THRIFTY) | DSHB | BA-F8-s | 1:1000 | 5 % NGS PBS-Tx |
| MHC II (THRIFTY) | DSHB | SC-71-s | 1:1000 | 5 % NGS PBS-Tx |
| Alexa-Fluor Goat Anti Mouse IgG2b 488 (THRIFTY) | ThermoFisher Scientific | #A-21141 | 1:1000 | 1 % NGS PBS-Tx |
| Alexa-Fluor Goat Anti Mouse IgG1 647 (THRIFTY) | ThermoFisher Scientific | #A-21240 | 1:1000 | 1 % NGS PBS-Tx |

CST; Cell Signaling Technology, DSHB; Developmental Studies Hybridoma Bank, SCBT; Santa Cruz Biotechnology, M-TBST; milk diluted in tris buffer saline with 1% tween, NGS; Normal Goat Serum, PBS-Tx; Phosphate buffered saline with 1% Triton-X.
